# High Expression of Plasma Extracellular HSP90α is Associated With the Poor Efficacy of Chemotherapy and Prognosis in Small Cell Lung Cancer

**DOI:** 10.3389/fmolb.2022.913043

**Published:** 2022-07-11

**Authors:** Baoyue Huang, Jinmiao Pan, Haizhou Liu, Yamei Tang, Shirong Li, Yingzhen Bian, Shufang Ning, Jilin Li, Litu Zhang

**Affiliations:** ^1^ Department of Research, Guangxi Medical University Cancer Hospital, Guangxi Medical University, Nanning, China; ^2^ Department of Research, Guangxi Cancer Molecular Medicine Engineering Research Center, Nanning, China

**Keywords:** SCLC-small cell lung cancer, eHSP90α, prognosis, response evaluation criteria in solid tumor, NSE

## Abstract

**Purpose:** eHSP90α is closely related to tumor progression and prognosis. This study aimed to investigate the significance of eHSP90α in the response evaluation and prediction of small cell lung cancer.

**Methods:** We analyzed the relationship between eHSP90α expression and clinicopathological features in 105 patients with small cell lung cancer. Univariate and multivariate analyses were used to determine the association of parameters and ratios with response assessment, progression-free survival (PFS), and overall survival (OS).

**Results:** In SCLC patients, eHSP90α and NSE were positively correlated. The cutoff values of eHSP90α in OS, PFS, and response evaluation were 61.2 ng/ml, 48.7 ng/ml, and 48.7 ng/ml, respectively. eHSP90α could better predict OS, PFS, and response evaluation (AUC OS 0.791, PFS 0.662, 0.685). Radiotherapy and eHSP90α were independent variables for effective chemotherapy through univariate and multivariate analysis. In contrast, radiotherapy, eHSP90α, NSE, and M stage were independent variables for OS. eHSP90α, and M stage were independent variables for PFS. Kaplan-Meier analysis showed that higher eHSP90α expression predicted poorer OS and earlier progression in patients.

**Conclusions:** This study aims to provide new evidence for the efficacy response and prognostic assessment of SCLC. eHSP90α may be a better biomarker for SCLC.

## Introduction

By 2021 and for the foreseeable future, it is commonly acknowledged in the academic and medical field that lung cancer is the most lethal type of cancer globally, and small cell lung cancer (SCLC) is a malignant tumor with a relatively poor prognosis (Mai et al., 2021). Most patients tend to be in a metastatic stage at the early diagnosis stage rather than a less aggressive stage (Pedersen et al., 2021). 30,000 to 35,000 new cases of SCLC, also known as oat cell carcinoma, is a neuroendocrine tumor derived from bronchial epithelial cells, which accounts for 13–15% of all lung cancers that appear in the US per year (Oronsky et al., 2017).Currently, techniques for diagnosis and response assessment of lung cancer involve image scanning, bronchoscopy, transbronchial needle aspiration, CT-guided puncture of lung tumor, etc. While comparatively low diagnostic sensitivity shows in image scanning, the level of invasiveness and undesirable effects also seem too apparent in the regular histological diagnostic procedure-bronchial biopsy. Subsequently, with possible early detection, accurate subtyping, and intensive monitoring being essential for an optimal result of SCLC therapy, the issue of SCLC being diagnosed at a later stage and drastically reducing curing rates of this aggressive malignancy will be at ease (Cummings et al., 2008; Fang et al., 2021).There are several potential markers carcinoembryonic antigen (CEA), Neuron-specific enolase (NSE), Progastrin releasing peptide (ProGRP), Creatine kinase BB (CK-BB), Chromogranin A (CgA), Neural cell adhesion molecule (NCAM) and several cytokeratins (Harmsma et al., 2013). NSE, also known as enolase-γ, is a neuro- and neuroendocrine specific isoenzyme of enolase which is frequently reported to show an elevation in SCLC at the time of diagnosis (Massabki et al., 2003; Tian et al., 2020). Notwithstanding, its levels are repeated tested to be correlated to tumor mass extension, for which it is considered to be a reliable marker while patients undergo chemotherapy, and it has proven to be a suitable biomarker for follow-up and a noteworthy predictor of SCLC patients (Zhou et al., 2017; Tian et al., 2020).

Heat shock protein 90 (HSP90) is a ubiquitously expressed molecular chaperone that plays an essential part in various biological processes with two isoforms in the cytosol, HSP90α (also known as HSP90AA1) and HSP90β (also known as HSP90AB1) (Li et al., 2013; Tian et al., 2019). HSP90α is an essential intracellular molecular chaperone for hundreds of diverse and important protein clients, making HSP90 a central regulator of cellular processes ranging from protein folding to DNA repair, development, immune response, and many other important functions (Hou et al., 2021). Previous studies showed that plasma Hsp90 levels are positively correlated with tumor malignancy, which may be a potential clinical diagnostic and prognostic marker (Wang et al., 2009). Moreover, extracellular HSP90α has recently been tested as an excellent biomarker for liver and lung cancer in clinical practices (Fu et al., 2017; Zhou et al., 2021). According to recent demonstrations, the area under the diagnosis curve of eHSP90α against lung cancers appears to be 0.857, with outstanding sensitivity and specificity. Thus, eHSP90α is well anticipated to be a game-changer in tumor biomarkers for lung cancer diagnosis and prognostic assessment (Zhou et al., 2021).

A nomogram is a graphical representation of a mathematical model involving several factors to predict a particular endpoint with statistical methods applied (Balachandran et al., 2015). Nomograms have been considered reliable tools to predict the clinical outcomes in several types of cancer and have proven to be more precise than the currently available staging systems (Iasonos et al., 2008). It is of certain urgency and great importance to developing a non-invasive methodology for observing small cell lung cancer prognosis. To allow improved monitoring of efficacy in therapeutic modalities and further accuracy in subtyping, we employed said the strategy to investigate the predictive value of various clinical variables and established a nomogram by analyzing SCLC patients.

## Methods and Materials

### Patients and Data Selection

105 patients diagnosed with small cell lung cancer (SCLC) were enrolled at the Department of Medical Oncology of Respiratory in Guangxi Medical University Cancer Hospital from January 2018 to April 2021. Ethics approval number: LW2022044.

**FIGURE 1:**
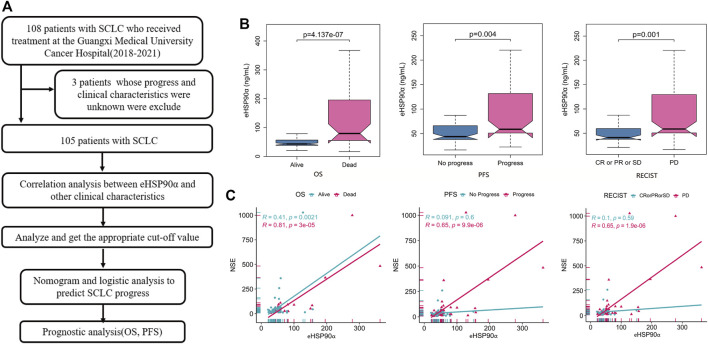
Workflow diagram and association of eHSP90α with clinical outcomes and NSE **(A)** Workflow **(B)** Box-plot shows the expression of eHSP90α in different outcome states of OS, PFS, and RECIST **(C)** Scatter plots display the relationship between two continuous variables, eHSP90α and NSE; different colors represent different OS, PFS, and RECIST states (NSE μg/L, eHSP90α ng/mL) Overall survival, OS; progression-free survival, PFS; Response Evaluation Criteria in Solid Tumors, RECIST.

SCLC patients were qualified for inclusion if they met the following criteria:1. They have pathologically diagnosed SCLC; 2. Obtained blood samples; 3. Response assessments were done with a computed tomography scan (CT scan) of the chest and abdomen every two treatment cycles according to Response Evaluation Criteria in Solid Tumors (RECIST version 1.1). Each patient’s response was classified into one of the following categories: responders, including cases of complete response (CR), partial response (PR), and stable disease (SD), and non-responders, including issues of disease progression (PD). The exclusion criteria were as follows: incomplete demographic statistical information such as age, sex, or race; incomplete clinicopathology information such as tumor size (defined as the most accurate measurement of a solid primary tumor), tumor-node-metastasis (TNM) stage, tumor grade; incomplete therapeutic information in chemotherapy and radiotherapy (defined as the method performed as part of the first course of treatment); missing survival status and follow-up data. According to the National Comprehensive Cancer Network (NCCN) combined with the Veterans Administration Lung Study Group (VALG) approach for SCLC staging, limited disease (LD)-SCLC is specified as stage I to III (T any, N any, M0), and extension disease (ED)-SCLC is defined as stage IV (T any, N any, M1a/b). All subjects involved had received treatment using etoposide plus platinum antineoplastic agent in the first place. For general methods, see Figure 1A.

In this retrospective study, the data on the peripheral blood of the patients diagnosed with SCLC were collected which included T lymphocytes, T helper cells, T suppressor cells, T helper cells/T suppressor cells, natural killer cells, B lymphocytes, carcinoembryonic antigen, carbohydrate antigen 19–9, thymidine kinase1, neuron-specific enolase and Ki-67. All blood samples were collected before subjects had received any treatment.

### Specimen Collection and Measurement of Tumor Markers

Heat shock protein 90α (Yantai Protgen Biotechnology Development Co., Ltd., Shandong, China): Collect blood from patients with EDTA-K2 anticoagulation tube in the morning and apply it in a centrifuge 3000RPM for 15 min. Incubate the kit in water at 37°C for 30 min. The liquid in the kit gets thoroughly mixed to a point where no air bubbles are visible. Add 4.75*10^5^ μL of deionized water to the concentrated washing solution and mix well. Add calibrator to 400 μL of analyte diluent to dissolve and mix. Plasma samples need to be diluted 20-fold with diluent. Take 50 μL each of the calibrator and the diluted plasma sample, add them to the calibration well and sample the microplate. Add 50 μL of heat shock protein 90α marker solution to the wells and mix gently. Subsequent incubation happens in warm water at 37°C for 60 min after being sealed. Shake off the reactant solution, add a 300 μL washing solution/washing machine, wash the plate six times, and dry on absorbent paper. Add 50 μL of chromogenic reagent A to the wells above, then add 50 μL of chromogenic reagent B and incubate with warm water at 37°C for 20 min after mixing. Add 50 μL of stopping solution to the above wells to stop color development. Read the OD value immediately at 450 nm/620 (630) nm wavelength. Calculate the content of HSP90α in plasma samples based on the software algorithm.

### Nomogram for OS, PFS, and Response Evaluation

The least absolute shrinkage and selection operator (LASSO) regression model selected the relevant factors for response evaluation and prognosis of small cell lung cancer. Logistic regression analysis was then used to build a convincing model for predicting chemotherapy efficacy and prognostic outcomes for small cell lung cancer by incorporating features selected in the LASSO approach. Clinicopathological features and biomarkers with *p*-values less than 0.05 in peripheral blood were included in the model. All selected predictors were used to develop a nomogram model to predict small cell lung cancer.

### Cell Culture

NCI-H146 and A549 lung cancer cells were purchased from the Institute of Cell Biology, Chinese Academy of Sciences (Shanghai, China). Cells were grown in Roswell Park Memorial Institute 1,640 medium containing 10% fetal bovine serum and 1% penicillin/streptomycin at 37°C in a humidified atmosphere with 5% CO_2_.

### Statistical Analysis

The data were expressed as median and quartile. The Spearman correlation analysis evaluated the association between eHSP90α and other indexes. Receiver operating characteristic (ROC) curves were used to identify optimal cutoff values of variables in overall survival, progression-free survival, and response to treatment. Grouped according to cutoff value, Least Absolute Shrinkage and Selection Operator (LASSO) was used to analyze eHSP90α and efficacy. R 4.03 software was used to draw Kaplan-Meier curves of OS and PFS. The univariate and multivariate Cox regression was performed to investigate the relationship between eHSP90α and OS and PFS. A *p-value* less than 0.05 was considered statistically significant. Statistical analyses were performed using the SPSS computer program (SPSS 22.0).

## Results

### SCLC Patients’ Characteristics and Hematologic Baseline

In total, 105 patients with SCLC were enrolled in our analysis. All patients were with an average age of 62.00 ± 8.55 years at the time of diagnosis. Of the 105 patients, 33 (31.43%) underwent radiotherapy and 94 (89.52%) underwent chemotherapy. 27 (25.7%) were classified with limited disease small cell lung cancer and 78 (74.3%) were extension disease small cell lung cancer. The other parameters clinicopathological data are shown in Table 1. Next, we analyzed the differences and progressive groups of all peripheral blood indicators in Table 1 Alive and DEAD groups in OS, progress and no progressive group in PFS and the differences between the CR \ PR \ SD and PD groups in Response Evaluation. We found that eHSP90α has different expressions in these groups, as shown in Figure 1B. The expression of other peripheral blood indicators in these groups, see Supplementary Figure S1.

**TABLE 1:** Patient characteristics and hematologic parameters.

Features	SCLC
Total	105
Gender	
Male (%)	87 (82.9%)
Female (%)	18 (17.1%)
Age (years)	
<55 (%)	23 (21.9%)
⩾ 55 (%)	82 (78.1%)
Location	
Left (%)	40 (38.1%)
Right (%)	60 (57.1%)
Mediastinal (%)	5 (4.8%)
Diameter(cm)	
≤3 (%)	26 (24.8%)
>3 (%)	79 (75.2%)
TNM stage	
Stage I (%)	3 (2.9%)
Stage II (%)	2 (1.9%)
Stage III (%)	22 (21.0%)
Stage IV (%)	78 (74.3%)
VALG stage	
LD-SCLC (%)	27 (25.7%)
ED-SCLC (%)	78 (74.3%)
T stage	
T1 (%)	14 (13.3%)
T2 (%)	17 (16.2%)
T3 (%)	19 (18.1%)
T4 (%)	55 (52.4%)
N stage	
0 (%)	6 (5.71%)
1 (%)	16 (15.24%)
2 (%)	37 (35.24%)
3 (%)	46 (43.81%)
M	
0 (%)	27 (25.7%)
1 (%)	78 (74.3%)
Chemotherapy	
Yes (%)	94 (89.52%)
No (%)	11 (10.48%)
Radiotherapy	
Yes (%)	33 (31.43%)
No (%)	72 (68.57%)
eHSP90α [median (IQR)]	54.90 (39.10.80.90)
Ki-67 [median (IQR)]	80 (70,90)
T lymphocytes [median (IQR)]	66.17 (60.20.72.30)
T helper cells [median (IQR)]	38.80 (33.20.45.90)
T suppressor cells [median (IQR)]	18.20 (15.60.23.40)
T helper cells/T suppressor cells [median (IQR)]	2.10 (1.60.2.90)
Natural killer cells [median (IQR)]	12.20 (9.20.18.60)
B lymphocytes [median (IQR)]	11.60 (8.10.15.80)
Carcinoembryonic antigen [median (IQR)]	4.38 (2.22.14.62)
Carbohydrate antigen 19–9 [median (IQR)]	10.50 (3.83.34.98)
Thymidine kinase1 [median (IQR)]	0.78 (0.41.1.54)
Neuron-specific enolase [median (IQR)]	30.81 (15.84.61.97)

Limited disease small cell lung cancer, LD-SCLC; extension disease small cell lung cancer, ED-SCLC.

### Correlations of eHSP90α and Clinical Index

NSE is currently a commonly used marker for SCLC, and eHSP90α is a new biomarker. The correlation between these two biomarkers and other clinical indicators is analyzed here. Correlation analysis revealed that eHSP90α was positively correlated to the diameter of the tumor and neuron-specific enolase (NSE) in SCLC patients. This analysis shows that eHSP90α was negatively related to T lymphocytes and T helper cells in SCLC patients. There is no significant relationship between eHSP90α and another clinical index in LD-SCLC patients. NSE and eHSP90α had the same trend in ED-SCLC patients **(** Table 2 **)**. NSE was positively correlated with eHSP90α in total SCLC and ED patients, indicating that eHSP90α is likely to have excellent diagnostic and prognostic efficacy. We next use the scattered dot diagram to show the relationship between the two continuous variables eHSP90α and NSE. We found that NSE and eHSP90α in the OS group, PFS progress group and PD groups in the Response Evaluation have significant correlations. See Figure 1C. Other correlation analysis results show in Supplementary Figures S1,S2.

**TABLE 2:** Correlation of eHSP90α and Other Index in SCLC Patients.

Parameter	Total		LD-SCLC		ED-SCLC	
	r	p	r	p	r	p
Sex	−0.090	0.363	−0.146	0.402	−0.110	0.366
Age	−0.087	0.378	0.145	0.407	−0.216	0.073
Diameter(cm)	0.213	0.029	0.165	0.342	0.223	0.064
T lymphocytes	−0.200	0.041	−0.404	0.016	−0.095	0.435
T helper cells	−0.199	0.042	−0.300	0.080	−0.148	0.222
T suppressor cells	−0.045	0.648	−0.262	0.128	0.048	0.691
T helper cells/T suppressor cells	−0.066	0.502	0.049	0.778	−0.102	0.399
Natural killer cells	0.129	0.191	0.273	0.112	0.050	0.682
B lymphocytes	−0.060	0.542	0.074	0.672	−0.054	0.660
Ki-67	0.021	0.831	0.286	0.096	−0.104	0.389
CEA	0.067	0.496	0.163	0.350	−0.020	0.867
CA19-9	0.146	0.138	0.196	0.260	0.112	0.359
TK1	−0.086	0.385	−0.147	0.401	−0.046	0.705
NSE	0.376	**0.001**	0.068	0.771	0.441	**0.001**

Spearman correlation, bold font indicates statistical. Carcinoembryonic antigen, CEA; Carbohydrate antigen 19–9, CA19-9; Thymidine kinase1, TK1; Neuron-specific enolase, NSE.

### The Cut-Off Value of eHSP90α Outcome Prediction Among SCLC Patients’ Index and Their Combined Prognostic Value

**FIGURE 2:**
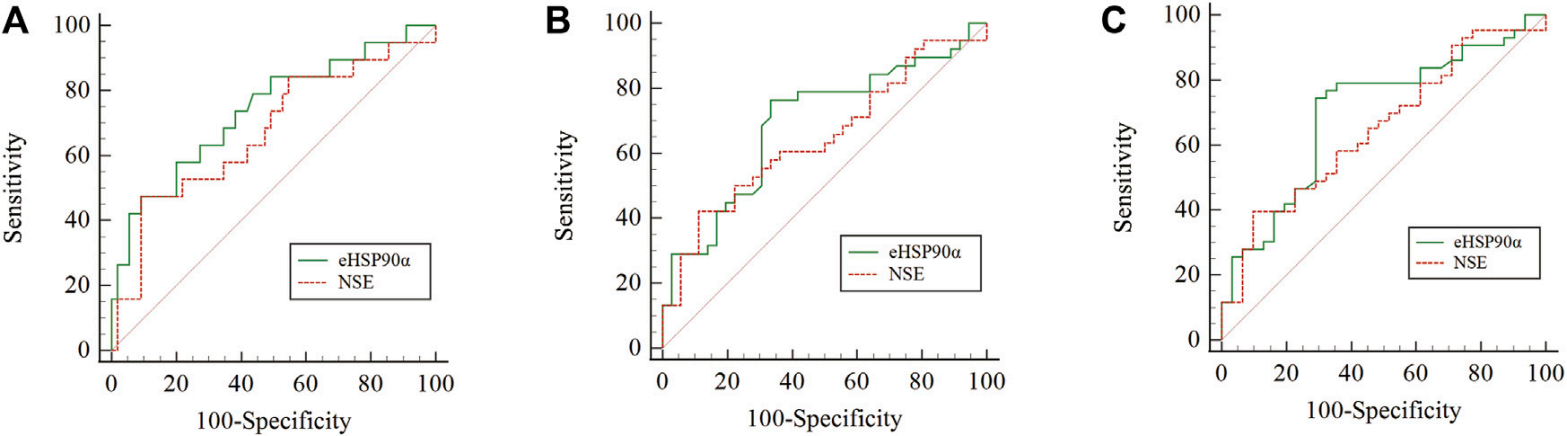
Comparison of the areas under the ROC for outcome prediction among the three status-based prognostic scores **(A)** OS status *p* = 0.3545 **(B)** PFS status *p* = 0.5394 **(C)** RECIST status *p* = 0.5246.

ROC curves were used to analyze the diagnostic effect of eHSP90α in dichotomous variables such as overall survival status (alive/dead), progression-free survival status (whether progress or recurrence) and response evaluation (RECIST version 1.1), see Table 3 eHSP90α had the highest AUC value (OS 0.791, PFS 0.662, response evaluation 0.685) in all three ROC curves. T helper cells/T suppressor cells had the highest specificity (0.984) in the OS ROC curve, and NSE had the highest specificity in the PFS ROC curve (AUC 0.889) and response evaluation ROC curve (AUC 0.903). T lymphocytes had the highest specificity (0.903) in the response evaluation ROC curve and the highest sensitivity (0.841) in the OS ROC curve. eHSP90α and T lymphocytes had the same highest sensitivity (0.792) in the PFS ROC curve. eHSP90α had the highest sensitivity (0.778) in the response evaluation ROC curve. eHSP90α had the highest Youden in the OS ROC curve (0.534) and response evaluation ROC curve (0.397), while T helper cells had the highest Youden (0.187) in the PFS ROC curve. Taken together, eHSP90α has better sensitivity and specificity than NSE, and similar diagnostic effectiveness as NSE in ROC curves of OS (Figure 2A), PFS (Figure 2B) and response evaluation (Figure 2C).

**TABLE 3:** Receiver operating characteristic curve of parameters.

Characteristics	OS					PFS					Response Evaluation				
	AUC (95%CI)	Threshold	specificity	Sensitivity	Youden	AUC (95%CI)	Threshold	specificity	Sensitivity	Youden	AUC (95%CI)	Threshold	specificity	sensitivity	Youden
B lymphocytes	0.53 (0.416–0.644)	8.265	0.787	0.341	0.128	0.526 (0.413–0.638)	7.93	0.865	0.302	0.167	0.504 (0.393–0.615)	13.45	0.714	0.429	0.143
CA199	0.523 (0.409–0.637)	4.25	0.311	0.791	0.102	0.552 (0.44–0.664)	6.55	0.431	0.717	0.148	0.629 (0.518–0.74)	6.55	0.524	0.758	0.282
CEA	0.553 (0.44–0.667)	9.355	0.721	0.442	0.163	0.607 (0.496–0.718)	3.86	0.725	0.585	0.31	0.538 (0.424–0.651)	3.855	0.667	0.5	0.167
eHSP90α	**0.791(0.698–0.884)**	61.2	0.852	0.682	**0.534**	**0.662(0.556–0.768)**	48.7	0.558	**0.792**	0.35	**0.685(0.58–0.79)**	48.7	0.619	**0.778**	**0.397**
Natural killer cells	0.631 (0.521–0.741)	11.75	0.541	0.705	0.246	0.512 (0.4–0.624)	11.23	0.442	0.66	0.103	0.565 (0.452–0.678)	11.225	0.5	0.683	0.183
NSE	0.673 (0.521–0.825)	68.55	0.909	0.474	0.383	0.646 (0.519–0.773)	57.33	**0.889**	0.421	0.31	0.65 (0.524–0.777)	57.325	**0.903**	0.395	0.299
T lymphocytes	0.61 (0.501–0.72)	70.35	0.443	**0.841**	0.284	0.555 (0.444–0.666)	72.10	0.346	**0.792**	0.139	0.626 (0.513–0.738)	68.82	0.595	0.667	0.262
T helper cells	0.577 (0.464–0.689)	30.95	0.918	0.25	0.168	0.59 (0.48–0.699)	32.2	0.885	0.302	**0.187**	0.616 (0.506–0.726)	42	0.571	0.635	0.206
T helper cells/T suppressor cells	0.499 (0.384–0.614)	3.8	**0.984**	0.091	0.075	0.577 (0.467–0.688)	2.05	0.635	0.528	0.163	0.565 (0.454–0.677)	2.05	0.643	0.508	0.151
TK1	0.565 (0.45–0.68)	0.46	0.77	0.386	0.157	0.487 (0.375–0.599)	0.54	0.692	0.358	0.051	0.525 (0.411–0.64)	0.615	0.69	0.429	0.119
T suppressor cells	0.456 (0.339–0.573)	23.765	0.787	0.295	0.082	0.527 (0.415–0.639)	18.15	0.538	0.566	0.104	0.496 (0.381–0.61)	18.15	0.548	0.556	0.103

Bold font indicates maximum value. Carcinoembryonic antigen, CEA; Carbohydrate antigen 19–9, CA19-9; Thymidine kinase1, TK1; Neuron-specific enolase, NSE.

### The Predictive Value of ehsp90α for the Effect of Chemotherapy in SCLC

**FIGURE 3:**
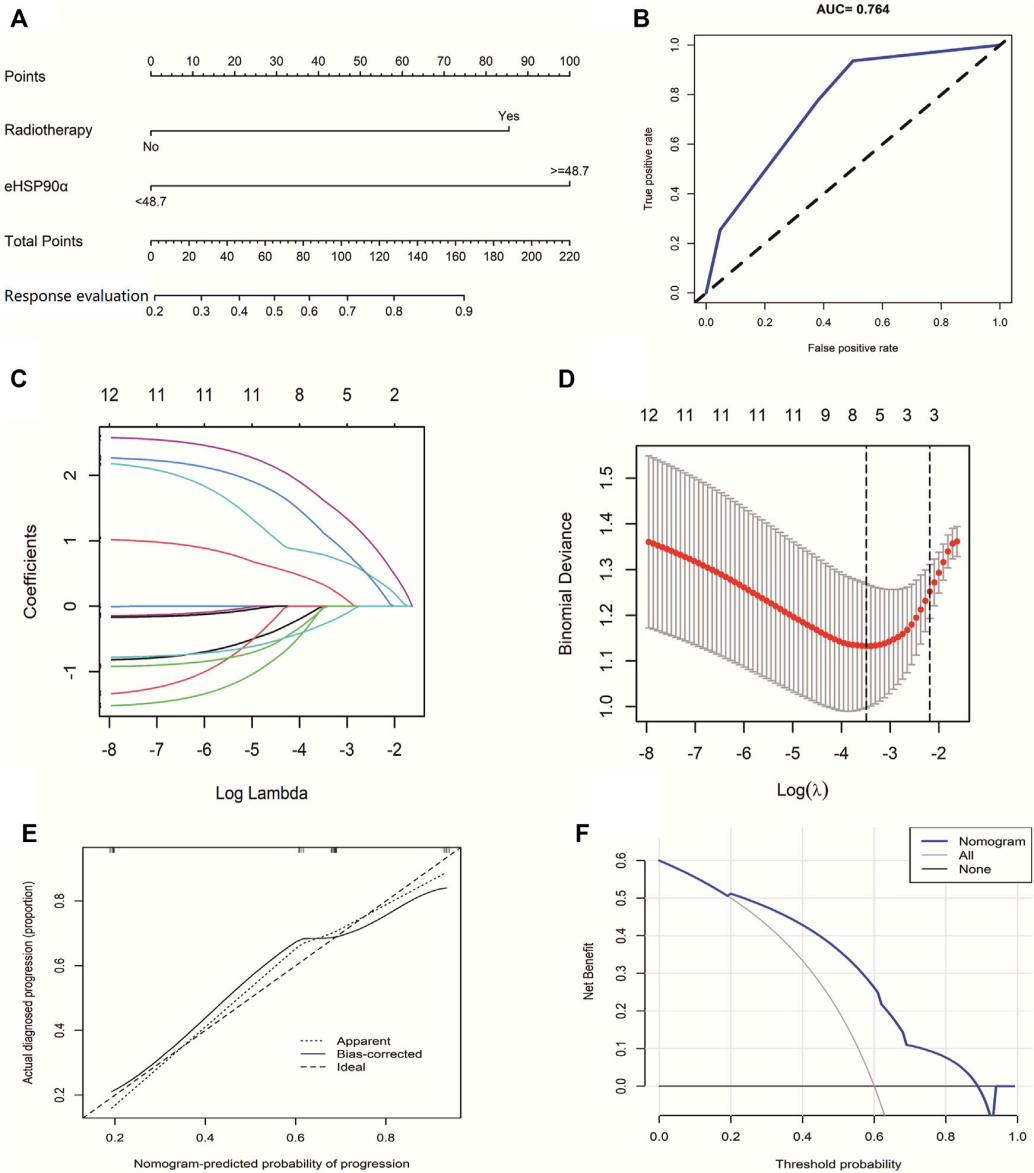
The Predictive Value of eHSP90α for SCLC effective chemotherapy **(A)** Established small cell lung cancer response evaluation nomogram with eHSP90α **(B)** Receiver operating characteristic curve of the model **(C)** LASSO coefficient profiles of the 12 features **(D)** A coefficient profile plot was produced against the logλ sequence **(E)** Calibration curves of the nomogram. Notes: The *x*-axis represents the predicted probability of progression. The *y*-axis represents the actual progression of small cell lung cancer. The diagonal dotted line represents a perfect prediction by an ideal model. The solid line represents the performance of the nomogram, of which a closer fit to the diagonal dotted line means a better prognosis **(F)** Decision curve analysis of our model showed that response evaluation nomogram. The x-axis is the risk threshold probability that changes from 0 to 1, and the y-axis is the calculated net profit for a given threshold probability.

Compared with OS and PFS, response evaluation (RECIST version1.1) does not contain information on survival time. Therefore, eHSP90α was used to explore the diagnostic significance of response status. We used two indicators, including eHSP90α, to establish a nomogram to evaluate the response of SCLC (Figure 3A). The receiver operating characteristic curve of the model had a higher AUC (0.764) than eHSP90α (AUC = 0.564) (Figure 3B). First, based on 105 SCLC patients, we used LASSO and 10-fold cross-validation to screen out six indicators (Figures 3C,D), including diameter, staging, chemotherapy, radiotherapy, location, eHSP90α (Figure 3C lambda. min = 0.03050932). To further select independent variables for effective chemotherapy from LASSO results, multivariate logistic regression analysis among these above features is shown in Table 4. The model finally contains radiotherapy (OR = 2.1103, *p* = 0.002276), eHSP90α (OR = 2.2677, *p* = 0.000524). The model was verified by bootstrapping (Figure 3E, C-index = 0.764). In this study, the threshold is from 20 to 88% (probability of patients and doctors); the decision curve analysis of our model showed that the response evaluation nomogram would profit more than of threshold (Figure 3F).

**TABLE 4:** Multivariate logistic regression analysis of influencing results of SCLC chemotherapy.

Variables	Multivariate Analysis		
	Odds Ratio	95% CI	*p*-value
Diameter (>3 cm)	0.606	5.321e-01–6.358e+00	0.333
Stage II	0.141	1.882e-30–5.250e+29	1.000
Stage III	16.417	6.130e-113-NA	0.994
Stage IV	17.368	7.726e-114-NA	0.994
Chemotherapy	−0.813	6.216e-02–2.630e+00	0.389
Radiotherapy	2.110	2.353e+00–3.692e+01	**0.002**
Right tumor	−0.662	1.587e-01–1.546e+00	0.249
Mediastinal tumor	−1.609	1.409e-02–2.533e+00	0.213
eHSP90α (≥48.7)	2.268	2.931e+00–3.997e+01	**0.001**

bold font indicates statistical.

### Clinical Prognostic Evaluation of eHSP90α in SCLC

**FIGURE 4:**
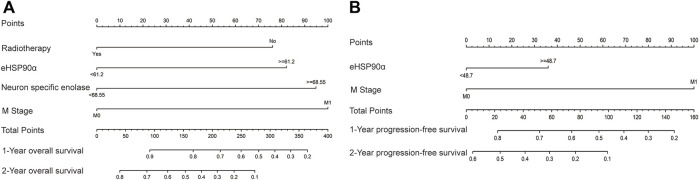
Nomograms of SCLC prognosis **(A)** The prognostic nomogram for overall survival (OS) based on the prognostic scores of eHSP90α and other factors in SCLC patients **(B)** The prognostic nomogram for progression-free survival (PFS) based on the prognostic scores of eHSP90α and M stage in SCLC patients.

**FIGURE 5:**
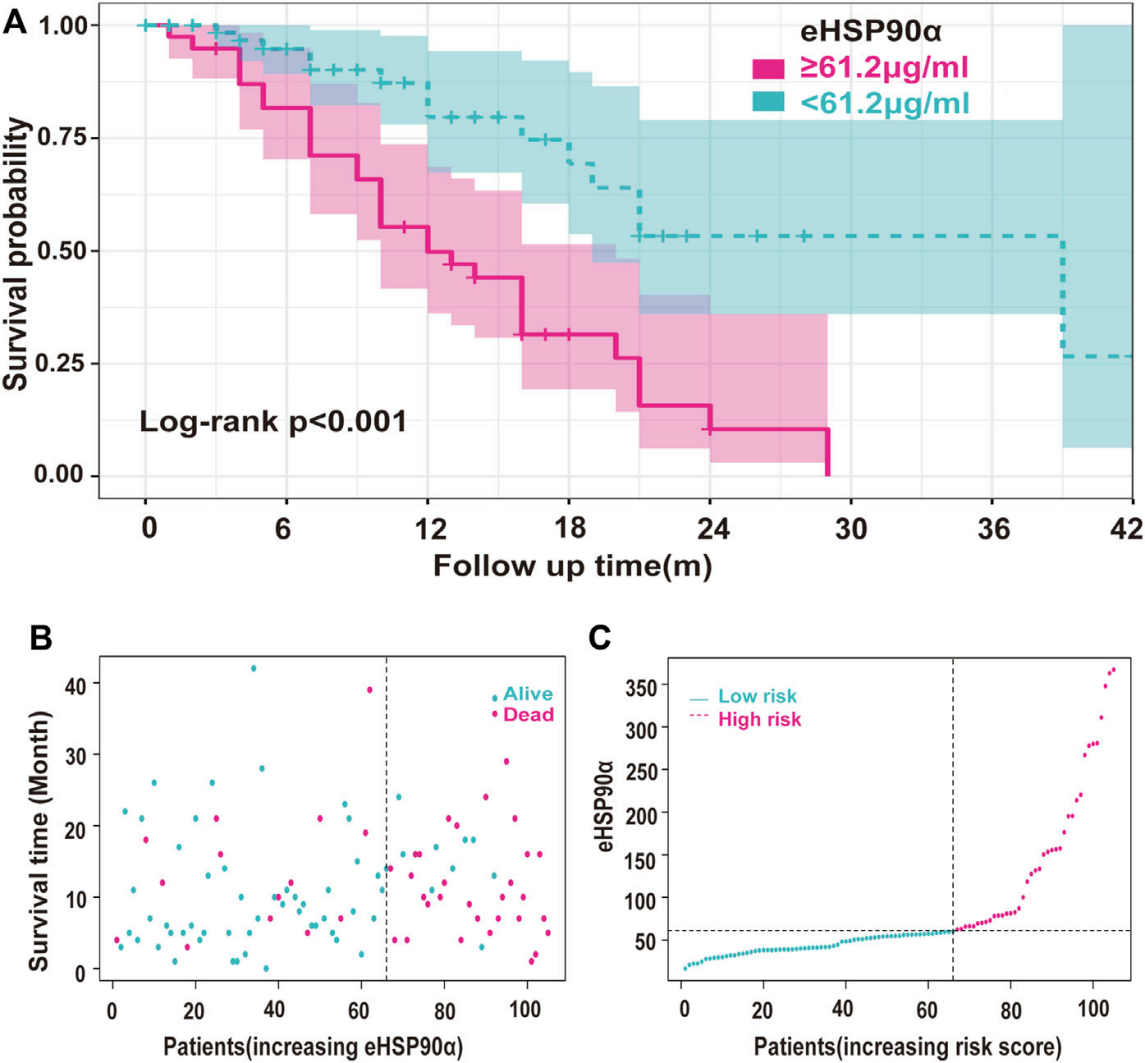
Survival analysis of the relationship between eHSP90α and OS in SCLC **(A)** Kaplan-Meier survival curves of the SCLC patients divided into two groups with 61.2 ng/mL as the cut-off value **(B)** Use the survival time model to build a scatter plot of risk score and survival time and color-coded said scatterplot. In contrast, red is for death, and blue is for survival based on the outcome **(C)** The survival time model was used to build a risk line graph, and patients are ranked high to low risks. The ordinate was the expression of eHSP90α, and the dotted line was the median value to distinguish between high and low stakes. Red indicates high risk, and blue indicates low risk.

**FIGURE 6:**
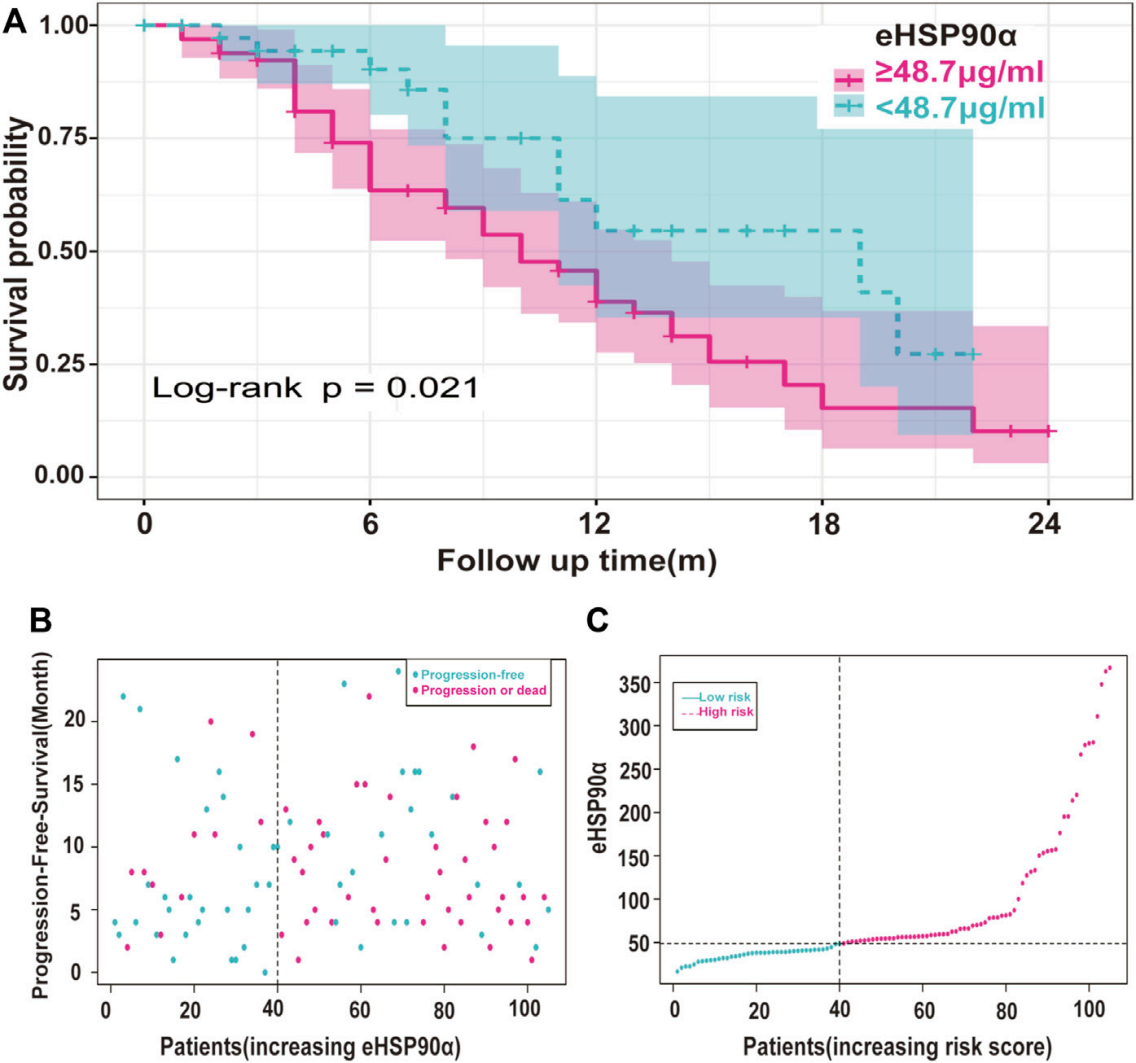
Risk Analysis of eHSP90α and progression in SCLC **(A)** Kaplan-Meier curves of the SCLC patients were divided into two groups with 48.7 ng/mL as the cut-off value **(B)** Scatter plots of risk score and time to progression were constructed using the survival time model and divided according to the results shown red for progression and blue for survival **(C)** A risk profile was created using a survival time model to rank patients from low to high risk. The ordinate is the expression of eHSP90α, and the dotted line is the median value, distinguishing high and low risks: red means high risk, and blue means low risk.

The ROC curve of the PFS was performed to determine the cutoff points of the index. The result is shown in Table 5. The threshold values of eHSP90α for OS, and PFS were 61.2 and 48.7, respectively. To determine which parameters were the independent prognostic factors of SCLC, univariate and multivariate analyses were conducted to explore the relationship between multiple variables, OS and PFS. In the multivariate analysis, high eHSP90α was an independent prognostic factor of a poor evolution in SCLC (OS, *p* < 0.001 and PFS, *p* = 0.024), whereas receiving radiotherapy was a good prognostic factor in SCLC (OS, *p* = 0.010 and PFS, *p* = 0.016). High NSE and M1 stage were significantly associated with shorter OS (NSE, *p* < 0.001 and M1 stage, *p* = 0.009). High CA-199 and M1 stage predicts earlier progression (CA-199, *p* = 0.018 and M1 stage, *p* < 0.001). We constructed a nomogram with four factors, including eHSP90α, to predict overall survival in SCLC patients (Figure 4A). The receiver operating characteristic curve of this model shows that AUC is 0.788. A nomogram for predicting progression-free survival was established with two indexes (Figure 4B). AUC is 0.671 in the receiver operating characteristic curve of this model. We analyzed the distribution of risk levels, survival status, and survival time patterns in SCLC patients divided into two groups with 61.2 ng/mL as the eHSP90α cut-off value. Kaplan-Meier survival analysis revealed that SCLC patients with higher eHSP90α expression had worse OS than those with lower eHSP90α expression (Figure 5A). Patients with high eHSP90α expression levels died more and survived shorter, as seen by survival time patterns (Figure 5B). The distribution of the eHSP90α risk score distribution suggests that higher expression of eHSP90α implies higher risk (Figure 5C). We analyzed the progression of SCLC patients and divided them into two groups with an eHSP90α cut-off value of 48.7 ng/ml. Kaplan-Meier survival analysis showed that the low expression group was more likely to be free of progression (Figure 6A). We made a scatterplot to assess progression risk, suggesting that more progression and higher mortality rates appear in the high-expression group. In contrast, patients’ conditions in the low-expression group were mostly non-progressive (Figure 6B). By sorting the patients according to the risk value from low to high, we obtained the progression risk curve graph, which shows the high expression of eHSP90α corresponds to a greater risk of progression (Figure 6C). eHSP90α above was significantly associated with NSE in ED-SCLC patients, but not in LD-SCLC patients. The occurrence of distant metastases is the main cause of poor PFS and OS in SCLC. But only eHSP90α but not NSE was significantly associated with poorer PFS in SCLC. We performed based on distant metastasis status for NSE and eHSP90α as we made a prediction that NSE might be associated with distant metastasis (Supplementary Tables S1,S2). The results showed that NSE was an independent factor affecting the distant metastasis of SCLC.

**TABLE 5:** Univariate and multivariate cox proportional regression analysis of OS and PFS in SCLC.

Variables	Univariate				Multivariate			
	OS		PFS		OS		PFS	
	HR (95%CI)	*p* Value	HR (95%CI)	*p* Value	HR (95%CI)	*p* Value	HR (95%CI)	*p* Value
Age (≤55 vs. >55)	1.610 (0.743–3.487)	0.227	1.022 (0.540–1.934)	0.948				
Chemotherapy (Yes vs. No)	0.431 (0.189–983)	0.045	2.321 (0.562–9.584)	0.244				
Diameter (≤3 cm vs. >3 cm)	1.610 (0.743–3.487)	0.227	1.957 (0.970–3.947)	0.061				
Ki-67 (≤50% vs. >50%)	2.572e+07 (0-Inf)	0.997	0.413 (0.099–1.717)	0.224				
M (M0 vs. M1)	2.562 (1.261–5.205)	**0.009**	5.326 (2.356–12.040)	**5.830e-05**	2.716 (1.323–5.573)	**0.006**	4.044 (1.777–9.204)	**0.001**
Mediastinal tumor	1.522 (0.348–6.661)	0.577	1.243 (0.290–5.332)	0.770				
N (N0 vs. N1-3)	3.159 (0.433–23.050)	0.257	N1-3 8.299e+07 (0-Inf)	0.996				
Radiotherapy (Yes vs. No)	0.398 (0.198–0.799)	**0.010**	0.467 (1.135–3.389)	**0.016**	0.403 (0.227–0.962)	**0.039**		
Right tumor	1.108 (0.591–2.077)	0.750	1.004 (0.5754–1.751)	0.990				
Stage (I-II vs. III-IV)	5.118e+07 (0-Inf)	0.997	8.433e-07 (0-Inf)	0.996				
T (T1-2 vs. T3-4)	0.881 (0.462–1.678)	0.699	1.422 (0.769–2.628)	0.262				
B lymphocytes (OS: < 8.27 vs. ≥8.27, PFS: < 7.93 vs. ≥7.93)	0.639 (0.341–1.201)	0.164	0.718 (0.398–1.295)	0.271				
CA-199 (OS: < 4.25 vs. ≥4.25, PFS: < 6.55 vs. ≥6.55)	1.650 (0.808–3.367)	0.169	2.268 (1.152–4.466)	**0.018**				
CEA (OS: < 9.36 vs. ≥9.36, PFS: < 3.86 vs. ≥3.86)	2.586 (1.406–4.758)	0.002	1.230 (0.6735–2.246)	0.501				
eHSP90α (OS: < 65.80 vs. ≥65.80, PFS: < 48.70 vs. ≥48.70)	3.410 (1.808–6.430)	**0.000**	2.155 (1.106–4.200)	**0.024**	2.271 (1.122–4.595)	**0.023**	2.056 (1.037–4.077)	**0.039**
Natural killer cells (OS: < 11.75 vs. ≥11.75, PFS: < 11.23 vs. ≥11.23)	1.785 (0.927–3.435)	0.083	1.130 (0.652–1.957)	0.664				
NSE (OS: < 68.55 vs. ≥ 68.55, PFS: < 57.33 vs. ≥57.33)	3.811 (1.872–7.758)	**0.000**	1.133 (0.659–1.949)	0.651	2.580 (1.200–5.546)	**0.015**		
T helper cells (OS: < 30.95 vs. ≥30.95, PFS: < 32.20 vs. ≥32.20)	1.010 (0.500–2.042)	0.977	0.852 (0.445–1.633)	0.630				
T helper cells/T suppressor cells (OS: < 3.80 vs. ≥3.80, PFS: < 2.05 vs. ≥2.05)	2.569 (0.914–7.221)	0.074	1.952 (0.700–5.441)	0.201				
T lymphocytes (OS: < 70.35 vs. ≥70.35, PFS: < 72.10 vs. ≥72.10)	0.489 (0.217–1.103)	0.085	0.874 (0.472–1.618)	0.668				
T suppressor cells (OS: < 23.77 vs. ≥23.77, PFS: < 18.15 vs. ≥18.15)	1.033 (0.538–1.986)	0.922	0.925 (0.502–1.706)	0.803				
TK1 (OS: < 0.46 vs. ≥0.46, PFS: < 0.54 vs. ≥0.54)	0.649 (0.351–1.198)	0.167	0.787 (0.435–1.424)	0.429				

bold font indicates statistical. Carcinoembryonic antigen, CEA; Carbohydrate antigen 19–9, CA19-9; Thymidine kinase1, TK1; Neuron-specific enolase, NSE.

### Validation of eHSP90α Expression in Supernatants of Two Lung Cancer Cell Lines

**FIGURE 7:**
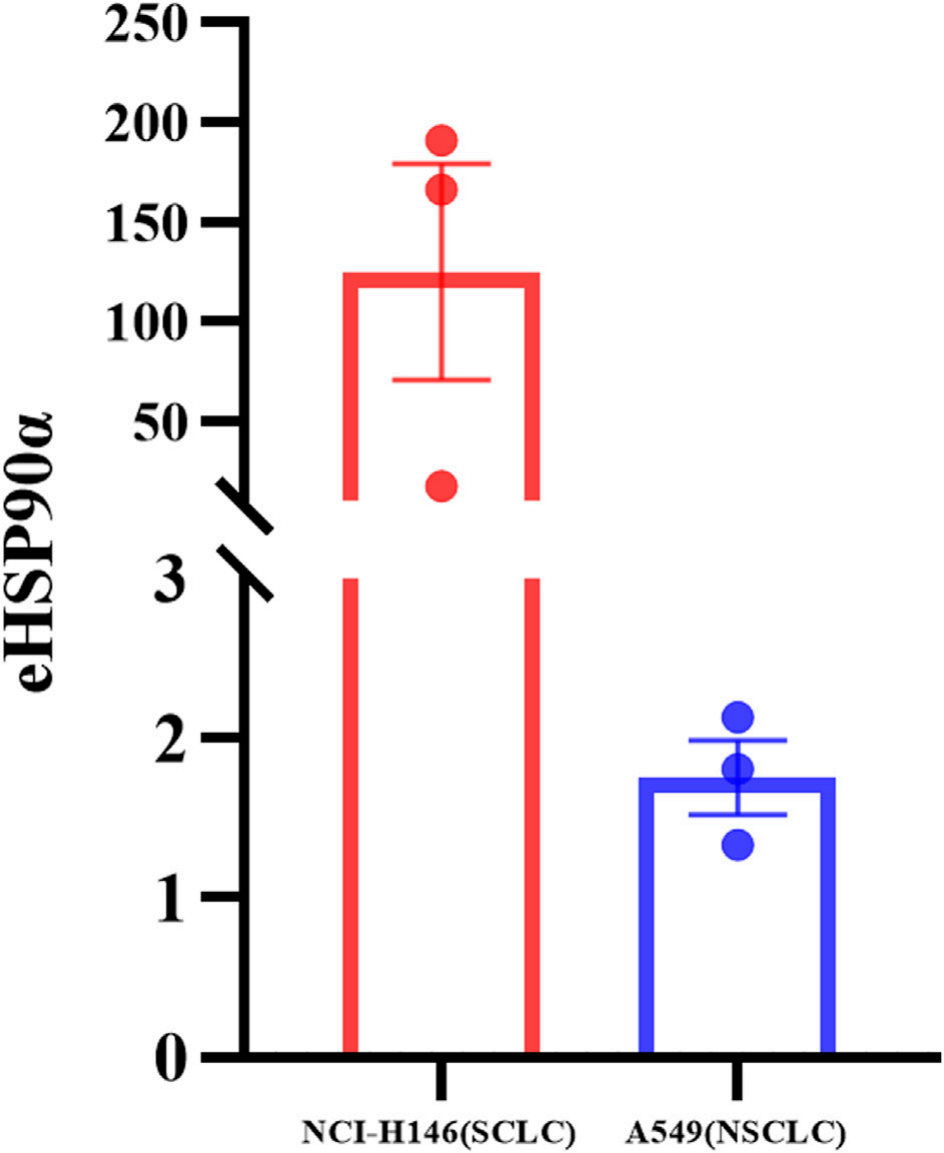
Differential expression of eHSP90α in SCLC and NSCLC cell lines. The expression of eHSP90α in NCI-H146 cell lines is higher than in A549 cell lines (*n* = 3).

To further explore the expression of eHSP90α in SCLC and NSCLC, we detected eHSP90α in different cell supernatants from six dishes (Supplementary Figure S3). The eHSP90α in SCLC cell supernatant is higher than in NSCLC (Figure 7). Consistent with our clinical results.

## Discussion

Small cell lung cancer prognosis has been challenging, especially in patients diagnosed during the ED (Wang et al., 2020). Some tumor markers have been used for the response assessment and prognosis monitoring, but they lack the level of sensitivity or specificity. In this study, the clinical index and the follow-up information of one hundred and five SCLC patients were analyzed to determine better markers.

Treatment of small cell lung cancer is very limited. In terms of response assessment, it has been shown that the neutrophil/lymphocyte ratio (NLR) value 6 weeks after anti-PD-1/PD-L1 antibody therapy appears to be a promising predictor of response in patients with small-cell lung cancer (Xiong et al., 2021). A study suggests that serum NSE and lactate dehydrogenase (LDH) may serve as biomarkers for predicting efficacy and survival in patients with small-cell lung cancer receiving first-line platinum-based chemotherapy (Liu et al., 2017). The ROC curve showed that the areas under the curve of NSE, ProGRP, and LDH expression on CR + PR were 0.683, 0.610, and 0.639, respectively, all of which were lower than those of eHSP90α.

In most studies’ prognosis of small cell lung cancer, NSE is a relevant tumor marker (Shibayama et al., 2001). NSE is often elevated in extensive-stage disease, and patients with high levels of NSE may suggest distant metastases, and higher NSE tends to predict shorter OS (Wang et al., 2021). The role of radiotherapy in SCLC has been controversial in the past, and the brain is SCLC’s most common metastatic site. Brain metastases occur in more than 50% of SCLC patients, and the prognosis is extremely poor, with an OS of only about 1–3 months. In the present study, eHSP90α exhibited similar diagnostic efficacy and prognostic value as NSE in patients with small-cell lung cancer. Prophylactic cranial irradiation (PCI) can reduce the occurrence of brain metastases. Several studies have confirmed that PCI can reduce the occurrence of brain metastases and improve overall survival in limited-stage patients with complete remission and extensive-stage patients with effective induction chemotherapy (Weaver and Coonar, 2017). However, a recent prospective clinical trial (Takahashi et al., 2017) from Japan showed that the incidence of brain metastases in the PCI group was 48 and 69% in the observation group, *p* < 0.001, but the 1-year OS did not improve (*p* = 0.094). Patients in our study who received radiotherapy at any site had longer OS. A study’s findings suggest that women have an advantage in survival compared to men (Cai et al., 2016). There were no statistically significant differences in the gender factor in our study, as the number of males was significantly higher than that of females in both studies. Therefore, the relationship between gender and prognosis is inconclusive. In addition, some studies suggest that the ratio of radiation dose to tumor diameter is related to the prognosis of limited-stage SCLC. Still, there is a lack of data on radiation dose in this study (Komatsu et al., 2010).

Studies have shown that NSE is an independent predictor of response and follow-up in SCLC, LD-SCLC, and ED-SCLC (Zhou et al., 2017). In our study, NSE and eHSP90α showed the same trend in all SCLC and a significant positive correlation in ED-SCLC. Serum NSE can also be present in platelets and red blood cells, and the results can be false-positive once the specimen has hemolysis or if the blood has been stagnant for a certain period (Massabki et al., 2003). Previous studies have found that the level of HSP90α in the serum of non-small cell lung cancer is significantly increased and it gradually increases with the clinical stage (Xu et al., 2007). The detection of a single tumor marker often has certain limitations, often cannot fully reflect the tumor, and has limited value for diagnosis and prognosis; therefore, the combined diagnosis of tumor markers can reduce the missed diagnosis rate of a single test and increase the accuracy of disease diagnosis.

Initially, 108 SCLC patients were included in the study while their medical records were being reviewed for information. Three patients were excluded due to missing data. In predicting OS, PFS, and response evaluation, eHSP90α had good AUC, sensitivity, and specificity. We built a nomogram from independent variables obtained from LASSO and multivariate logistic regression analysis. The AUC of this model is 0.764. Of the 105 SCLC patients who were finally included in the study, 39 patients had elevated eHSP90α expression (>61.2 ng/ml), and 66 patients had eHSP90α below 61.2 ng/ml. Through survival analysis, we found that patients with elevated eHSP90α expression had a higher risk of death than those with low expression. This is consistent with previous studies. We found that the expression of eHSP90α in SCLC cells was higher than that in NSCLC cells, which more experiments should confirm. We still need to investigate the mechanism further.

Due to the low proportion of small cell lung cancer in lung cancer, the sample size of this study is limited. The next step will be to expand the validation trial with a more rigorous sample size and conduct a long-term follow-up to determine the rigid prognosis. eHSP90α is a novel biomarker. Currently, there are few commercial kits to choose from. When more detection methods are available in the future, we will carry out strict verification through other detection methods.

In conclusion, this study found that SCLC patients with eHSP90α overexpression had a worse response and survival times. Diameter, staging, chemotherapy, radiotherapy, location, and eHSP90α have significant effects on patient response evaluation. eHSP90α and radiotherapy were independent factors in response evaluation in SCLC patients. eHSP90α, NSE, M staging, radiotherapy, and CEA had significant effects on OS in SCLC. Among them, eHSP90α, NSE, M stage, and radiotherapy were the independent prognostic factors affecting the survival of patients. This study aims to provide new evidence for the efficacy response and prognostic assessment of SCLC. eHSP90α may be a better biomarker for SCLC.

## Data Availability

The original contributions presented in the study are included in the article/Supplementary Material, further inquiries can be directed to the corresponding authors.

## Abbreviations

eHSP90α, extracellular heat shock protein 90 aa1; SCLC, Small cell lung cancer; NSCLC, non-small cell lung cancer; OS, overall survival; PFS, progression-free survival; AUC, area under the curve; NSE, neuron-specific enolase; CT, computed tomography; CEA, carcinoembryonic antigen; ELISA, Enzyme-linked immunosorbent assay; RPM, Round per minute; DI water, Deionized water.
