# Supplementary material for: High Expression of Plasma Extracellular HSP90α is Associated With the Poor Efficacy of Chemotherapy and Prognosis in Small Cell Lung Cancer
Source: Front Mol Biosci. 2022 Jul 11;9:913043. doi: 10.3389/fmolb.2022.913043 (PMC9309551; doi:10.3389/fmolb.2022.913043)
Supplement: Supplementary file 1 [file DataSheet1.docx]

***Supplementary Material***

***
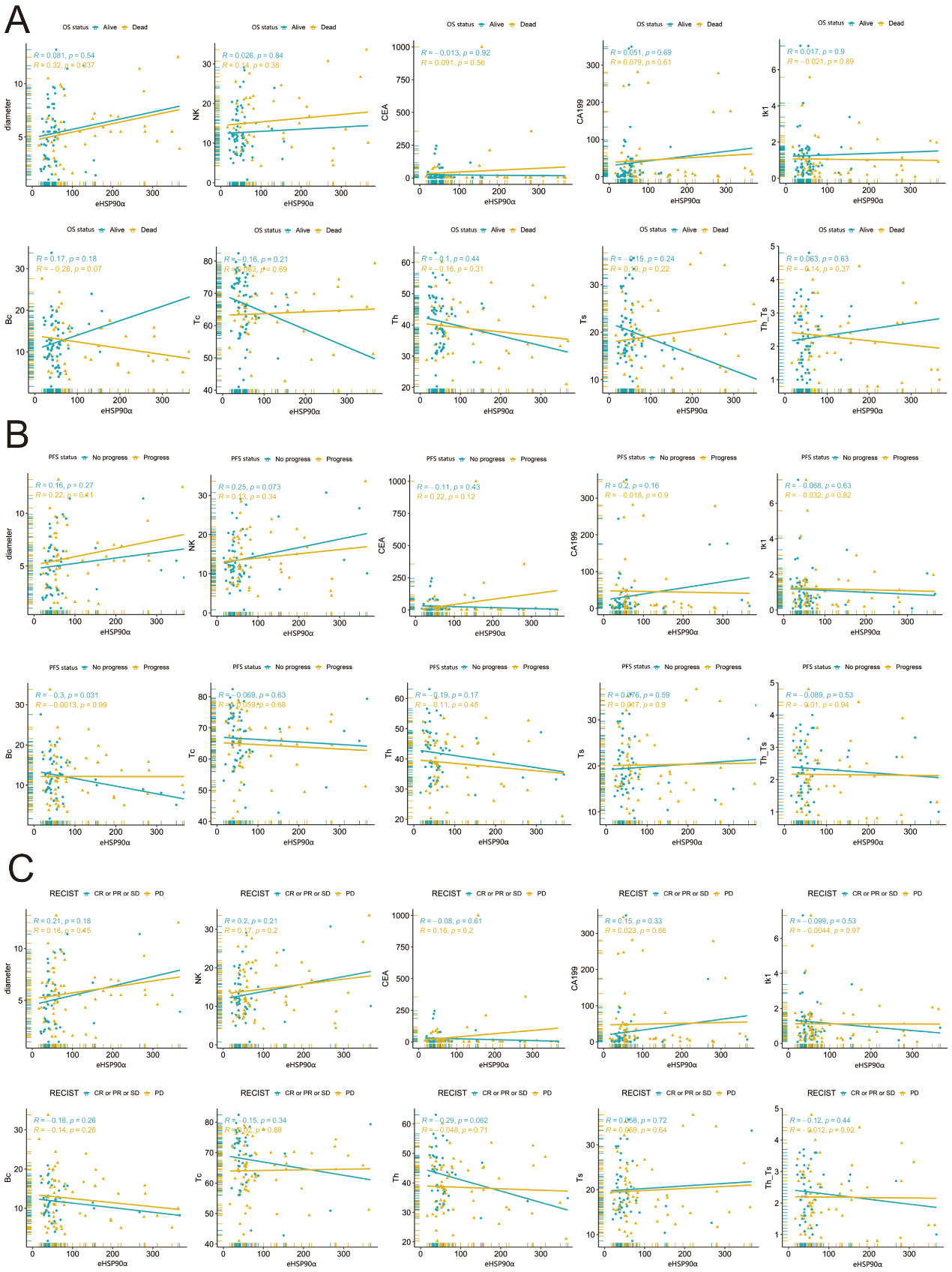
***

**Supplementary Figure S1.** **Scatter plot of the relationship between eHSP90α and continuous variable clinicopathological characteristics.** Scatter plots are used to display the relationship between two continuous variables eHSP90α and B cell, CA199, CEA, diameter, NK cell, Tc, Th, Ts, Th/Ts, tk1, different colors represent different OS, PFS and RECIST states. (A) OS. (B) PFS. (C) RECIST.

**
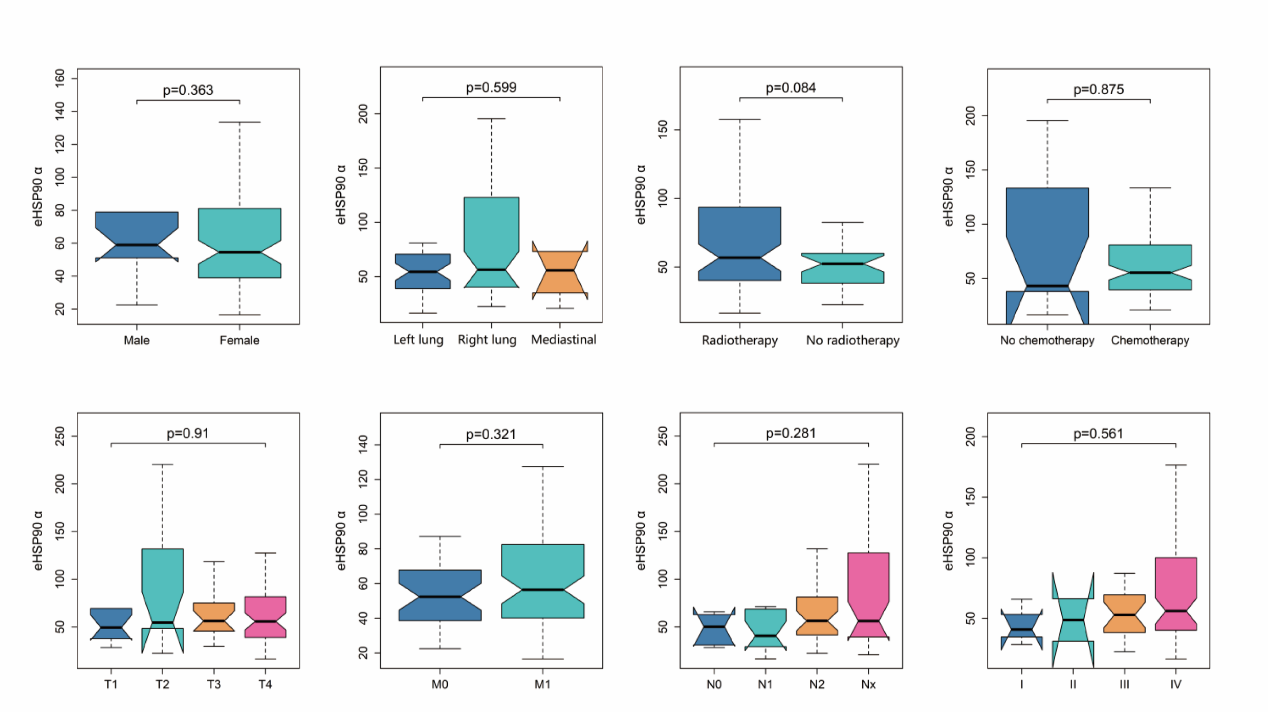
**

**Supplementary Figure S2. Correlations of the expression levels of eHSP90α with gender, tumor stages, tumor site, and prognosis of radiation and chemotherapy in small cell lung cancer patients.** There is no significant correlation between the above indicators and the expression level of eHSP90α.

**
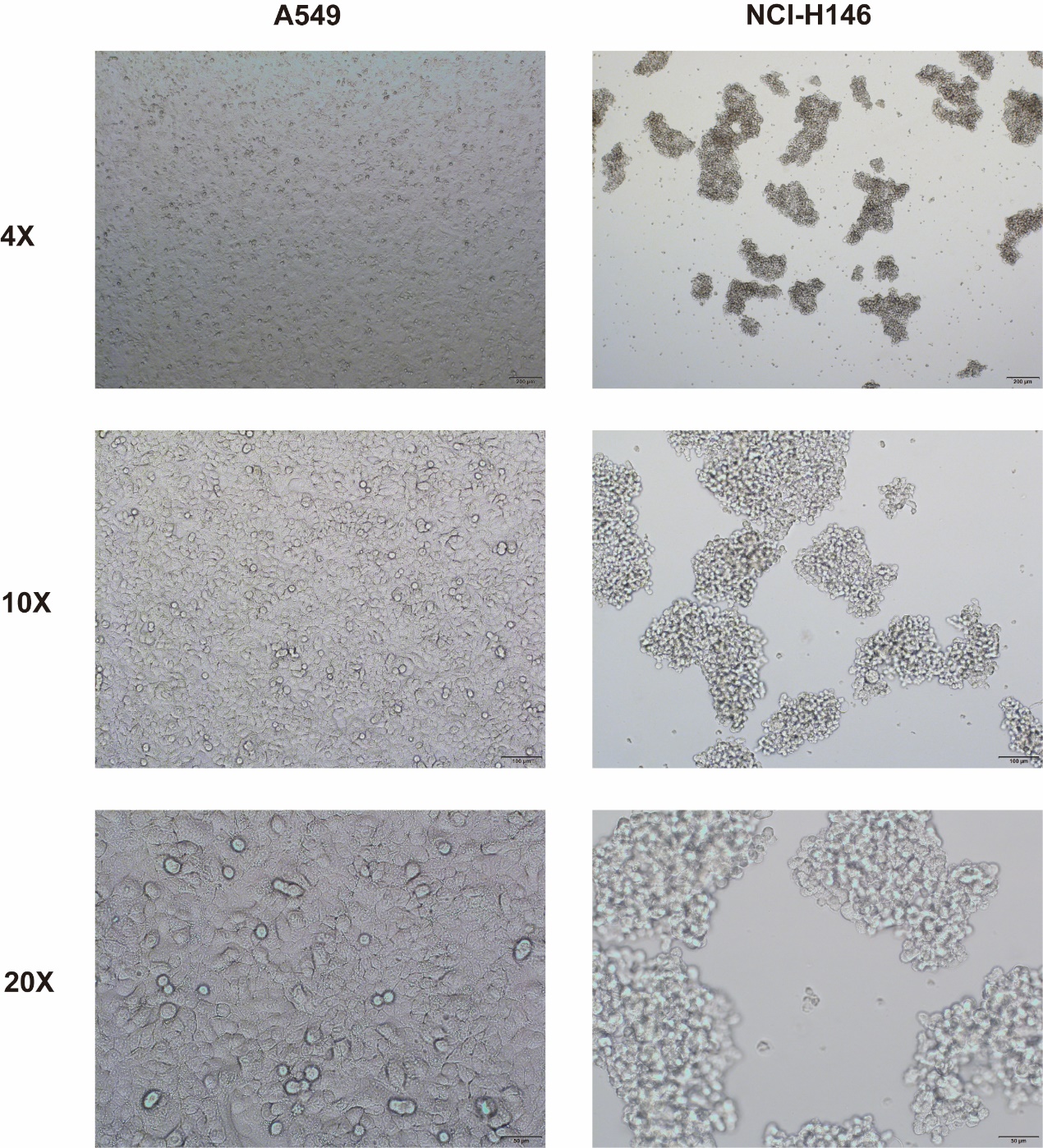
**

**Supplementary Figure S3.** **The morphology of A459 and NCI-H146 under 4×, 10×, 20× magnification.** Cell A459 on the left, NCI-H146 cell on the right.

**Supplementary methods**

Cell culture protocol: A549 cell line: When 80-90% confluency is reached, cells are washed and passaged with phosphate buffered saline (PBS) (11 mM KH2PO4, 26 mM Na2HPO4, 115 mM NaCl, pH 7.4). NCI-H146 cell line: When the cell density reaches 80-90%, the cell pellet is collected by centrifugation, and the cells are resuspended in fresh medium for passage. Medium was changed every 2 days and cells were passaged every 6-7 days by splitting in the ratio 1:3. After culturing the above cells for 48 hours, 10 μL of cell supernatant was collected from each flask for HSP90α assay.

**Supplementary Table 1 Univariate Logistic Regression Analysis of M stage in SCLC.**

| **Variables** | **Univariate analysis** | | |
| --- | --- | --- | --- |
|  | **Odds ratio** | **95 % CI** | ***p*-value** |
| NSE | 1.04 | 1.000-1.086 | **0.046** |
| eHSP90α (≥48.7) | 1.004 | 0.997-1.010 | 0.2474 |

**Note: bold font indicates statistical.**

**Supplementary Table 2 Multivariate Logistic Regression Analysis of M stage in SCLC.**

| **Variables** | **Multivariate analysis** | | |
| --- | --- | --- | --- |
|  | **Odds ratio** | **95 % CI** | ***p*-value** |
| NSE | 1.039 | 1.008-1.087 | **0.048** |
| eHSP90α (≥48.7) | 0.998 | 0.976-1.024 | 0.882 |

**Note: bold font indicates statistical.**
